# Case report: Successful combination of CLL1 CAR-T therapy and hematopoietic stem cell transplantation in a 73-year-old patient diagnosed with refractory acute myeloid leukemia

**DOI:** 10.3389/fimmu.2024.1454614

**Published:** 2024-09-17

**Authors:** Yifan Zhao, Hao Wang, Yu Zhang, Yi Zhang, Xiaomei Zhang, Mohan Zhao, Jile Liu, Shujing Guo, Mingfeng Zhao

**Affiliations:** ^1^ The First Central Clinical College of Tianjin Medical University, Tianjin, China; ^2^ Department of Intensive Care Medicine, Tianjin Hospital, Tianjin, China; ^3^ Department of Hematology, Tianjin First Central Hospital, Tianjin, China; ^4^ Nankai University School of Medicine, Tianjin, China

**Keywords:** acute myeloid leukemia, CLL1, chimeric antigen receptor T cell, hematopoietic stem cell transplantation, older

## Abstract

The incidence of Acute myeloid leukemia (AML) increases with advancing age, and the prognosis for elderly patients is significantly poorer compared to younger patients. Although the combination therapy of venetoclax and hypomethylating agents has demonstrated improved prognosis in patients unable to tolerate intensive chemotherapy, there remains a therapeutic blank for those who fail to achieve remission with current treatment regimens. Here, we report the successful clinical utilization of autogenous CLL1 CAR-T therapy combined with hematopoietic stem cell transplantation in a 73-year-old patient diagnosed with refractory AML. The patient achieved morphological complete remission (CR) with incomplete marrow recovery and a slight presence of minimal residual disease (MRD) after receiving CLL1 CAR-T therapy. To further enhance the treatment and promote the recovery of hemopoiesis, we performed bridged allogenic hematopoietic stem cell transplantation (allo-HSCT) 20 days after the infusion of CLL1 CAR-T cells. The patient achieved MRD-negative CR following HSCT treatment. His primary disease maintained a complete remission status during the 11-month follow-up period. The patient encountered grade 2 cytokine release syndrome and grade 4 granulocytopenia subsequent to the infusion of CAR-T cells, while several rounds of infection and graft-versus-host disease were observed following allo-HSCT. Nevertheless, all these concerns were successfully addressed through comprehensive provision of supportive treatments. We have successfully demonstrated a highly effective and safe combination strategy involving CLL1 CAR-T therapy and allo-HSCT, which has exhibited remarkable tolerability and holds great promise even for elderly patients with AML.

## Introduction

Acute myeloid leukemia (AML) is a highly aggressive neoplastic proliferation that affects the hematopoietic system, exhibiting an increased incidence beyond the age of 50, with a median age at diagnosis of 68 years (1, 2). In contrast to younger individuals, older patients diagnosed with AML exhibit poorer physical condition, a higher occurrence of coexisting medical conditions, and an increased proportion of unfavorable prognostic factors such as adverse cytogenetics or secondary acute myeloid leukemia (3). The majority of elderly patients are not eligible for aggressive chemotherapy (4). The current combination therapy of venetoclax and hypomethylating agents (HMAs) has demonstrated a remarkably high remission rate, with 67% achieving complete remission, thereby significantly prolonging patient survival (5, 6). However, the patients who were unable to attain remission through this treatment are devoid of other further therapeutic alternatives. Recently, the utilization of Chimeric Antigen Receptor T-cell (CAR-T) therapy has progressively increased in the treatment of hematological malignancies. The effectiveness and safety of CLL1 CAR-T therapy have led to its widespread acceptance in the treatment of patients with refractory/relapsed AML (7, 8).

Here, we reported a 73-year-old male who refractory to intensive chemotherapy, and the combination treatment of venetoclax and HMAs, but achieved complete remission from CLL1 CAR-T therapy, and he successfully received allogenic hematopoietic stem cell transplantation (allo-HSCT) for consolidating efficacy. Our study aimed to propose a potential combination regimen of CLL1 CAR-T therapy followed by HSCT for elderly patients with refractory/relapsed AML.

## Case presentation

**Figure 1:**
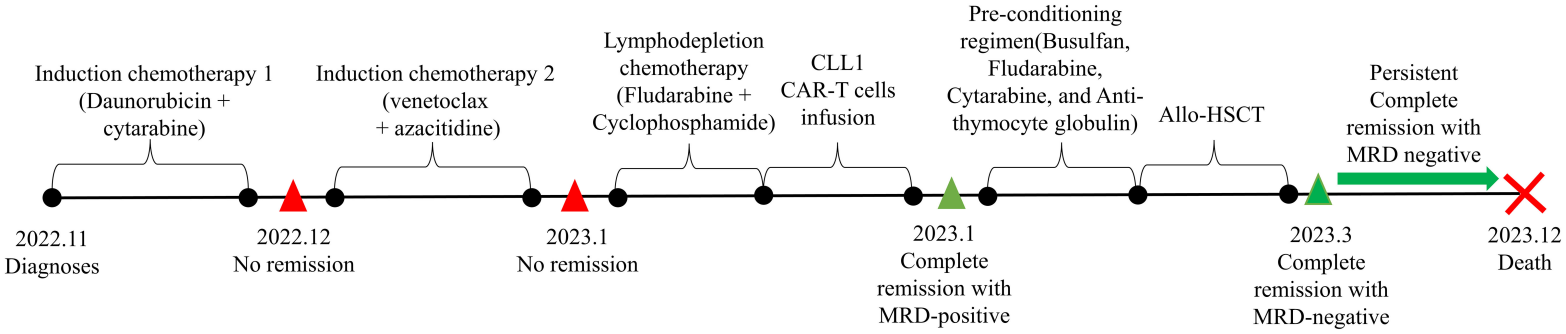
The timeline of patients’ treatment and efficacy evaluation. CAR-T, Chimeric Antigen Receptor T-cell; Allo-HSCT, allogenic hematopoietic stem cell transplantation; MRD, minimal residual disease.

The patient, a 73-year-old male, presented at our hospital in November 2022 with markedly elevated levels of white blood cells (WBC) at 231.41*109/L. He was diagnosed with acute myeloid leukemia and classified into the high-risk group based on the molecular genetics analysis, which revealed the presence of FLT3-ITD and TET2 mutation. Additionally, the cellular genetics analysis indicated a normal karyotype. Following tumor reduction prechemotherapy with hydroxycarbamide and low dose cytarabine, the patient underwent induction chemotherapy consisting of daunorubicin and cytarabine. However, the recheck conducted 14 days after chemotherapy completion showed residual blasts in bone marrow at a rate of 19.48%. Therefore, he underwent the second round of low-intensity chemotherapy with venetoclax and azacitidine. However, unfortunately, remission was still not achieved as the blasts percentage in the bone marrow increased to 79.52% at the third bone marrow morphological examination. The complete treatment process and evaluation of remission for this patient were illustrated in **Figure 1**.

**Figure 2:**
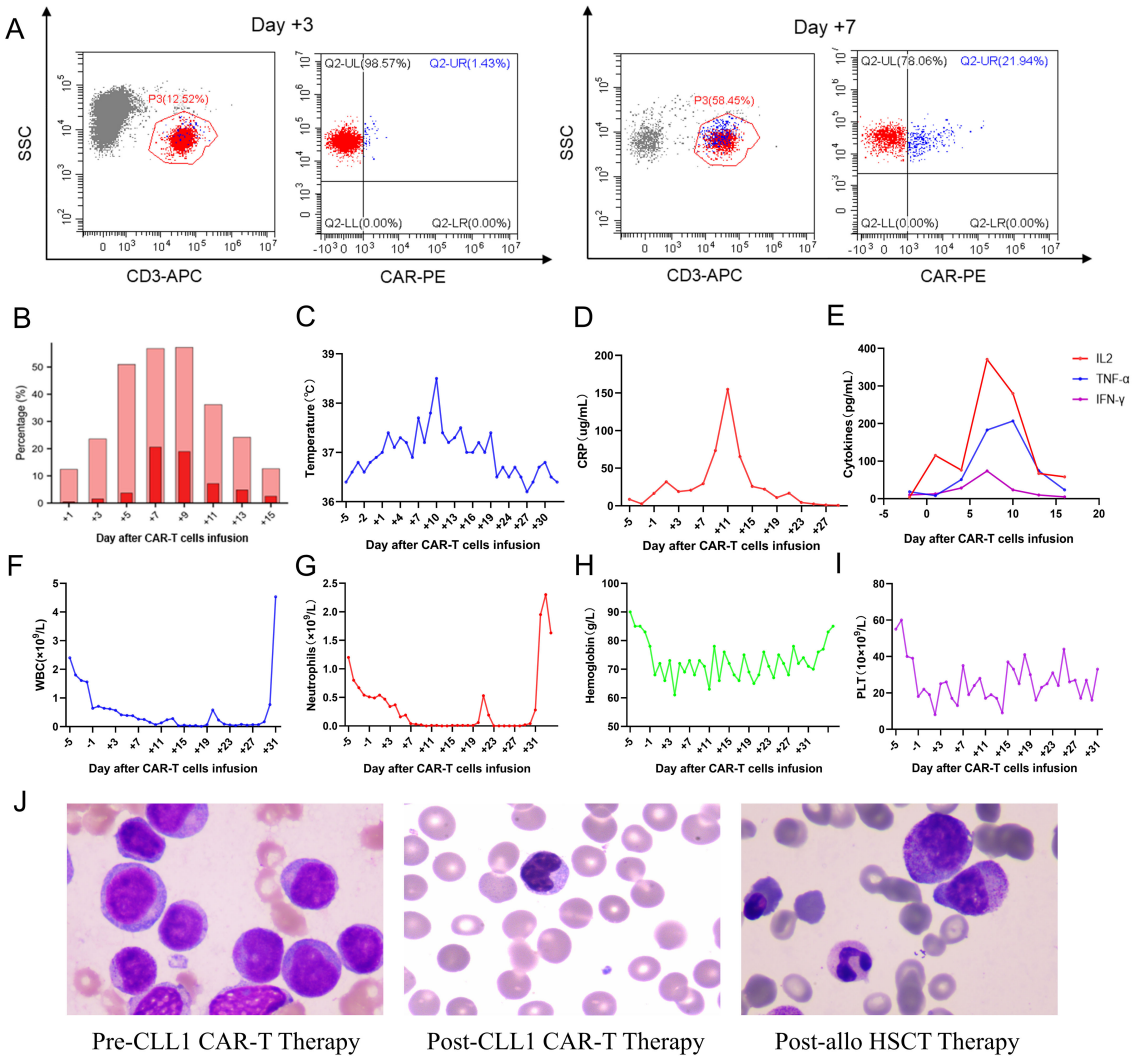
Clinical data measurements during the infusion of CAR-T cells. **(A)** Cytometric diagrams depicting the expansion of CAR-T cells within peripheral CD3-positive cells on Day +3 and +9; **(B)** CAR-T cells expansion variation in peripheral blood; **(C-E)**. The temperature peak, C-reactive protein, and cytokine profiles including IL6, TNF-α, and IFN-γ assessment during the CAR-T cell therapy and allo-HSCT treatment; **(F-I)**. The white blood cells (WBC), neutrophils, hemoglobin, and platelets (PLT) variation during the combination therapy; **(J)** The morphological detection at pre-lymphodepletion, post-CAR-T cell infusion, and post-HSCT.

Considering the overexpression of CLL1 on the surface of blasts in this patient, he and his family members have made a decision to participate in the clinical trial for CLL1 CAR-T therapy targeting refractory/relapsed AML, which was conducted at Tianjin First Centre Hospital (ChiCTR2000041054). The structure of the CLL1 CAR consists of a scFv derived from CLL1, a CD8 hinge and transmembrane domain, a 4-1BB costimulatory signal, and a CD3ζ intracellular signal domain. Additionally, it incorporates an innovative compact marker/suicide gene (RQR8) that combines target epitopes from both CD34 and CD20 antigens. This unique design allows for labeling with a CD34 antibody and elimination using Rituximab. The CLL1 CAR-T cells were manufactured at the Laboratory of Hematology Department in Tianjin First Central Hospital, involving T cell isolation, activation, lentivirus transfection, T cell expansion and culturing, transfection rate detection, cellular quality control measures, cell infusion procedures, and post-infusion monitoring. The transfection efficiency of CLL1 CAR-T cells was 45.02% at day 2 post lentivirus transfection, and 46.23% on the day of CAR-T cell infusion. Subsequently, the patient underwent a 3-day lymphodepletion chemotherapy regimen, comprising of Fludarabine administered at a daily dosage of 50mg and Cyclophosphamide administered at a daily dosage of 0.6g, followed by an infusion of autogenous CLL1 CAR-T cells. The dosage of CAR-T cells infused into his body was calculated as 5.2×105 CAR-T cells per kilogram of body weight. After the infusion of CAR-T cells, the patient experienced intermittent fever with a peak body temperature of 38.5°C. Meanwhile, we investigated the expansion of CAR-T cells during treatment, and observed that the peak proportion of CAR-T cells in peripheral blood CD3 cells occurred on day 7, reaching 21.94% (**Figures 2A, B**). Additionally, the temperature and inflammatory markers including IL6, IFN-γ, TNF-α, and C-reactive protein (CRP) also reached their highest levels on days 7-10 (**Figures 2C-E**). During the treatment, he experienced grade 1 cytokine release syndrome (CRS) and promptly recovered following tocilizumab intervention, with no observed neurological toxicities. The patient exhibited significant hematological toxicities, including granulocytopenia, anemia, and thrombocytopenia, predominantly starting from day 2-3. The changes in blood cell counts were illustrated in **Figures 2F-I**.

The patient achieved morphological complete remission with incomplete marrow recovery following CLL1 CAR-T therapy. However, more precise flow cytometry revealed a residual presence of 2.58% tumor cells in the bone marrow. Therefore, to eliminate the MRD, minimize the risk of relapse, and facilitate neutrophil recovery, the patient underwent subsequent bridged allo-HSCT after a 20-day interval following CAR-T cell infusion. After the administration of a pre-conditioning regimen consisting of busulfan, fludarabine, cytarabine, and anti-thymocyte globulin, the patient underwent infusion of donor-derived stem cells (CD34 positive cells: 3.92×10^6^/Kg; mononuclear cells: 11.32×10^8^/Kg). The prophylaxis of graft-versus-host disease (GVHD) involved the utilization of tacrolimus, mycophenolate mofetil, and methotrexate. The successful engraftment of the granulocyte and megakaryocytic series was accomplished on day 11 following the infusion of stem cells. The morphological detection variation of this patient was illustrated in **Figure 2J**, showcasing the disease status at multiple time points including pre-lymphodepletion, post-CAR-T cell infusion, and post-HSCT. The patient subsequently experienced multiple episodes of infection, as well as cutaneous and enteric mild GVHD, which were effectively managed through adjustments in anti-infective and anti-GVHD therapy. Despite the successful control of the primary ailment and persistent absence of minimal residual disease during subsequent monitoring, regrettably, the patient succumbed to an untimely demise due to sudden cardiac arrest after 11 months of CLL1 CAR-T therapy.

## Discussion

The prognosis of elderly patients with AML was significantly inferior to that of younger patients, which was influenced by multiple factors. The prognosis data of AML patients over the past 50 years, as analyzed by Kantarjian et al., revealed a modest increase in the 5-year survival rates for patients aged above 60, rising from a mere 8% in the 1970s to approximately 17% in the 2010s. In contrast, younger patients experienced a significant improvement with their survival rates increasing from only 13% in the 1970s to an impressive rate of 55% in the 2010s (9). The prognosis of elderly patients is influenced by both host factors and disease factors. On one hand, elderly patients exhibit reduced tolerance towards intensive chemotherapy due to a higher likelihood of complications and deteriorating physical conditions. On the other hand, patients over 60 years old commonly present with high-risk chromosomal abnormalities and worse cytogenetics (10). Additionally, older patients also have a higher occurrence of multidrug resistance, which rises up to 57% in individuals above 70 years old (11). Therefore, there was an urgent need to identify more conservative, tolerable yet effective treatments for elderly patients in order to enhance prognosis and minimize treatment-related mortality.

The recent combination regimen of venetoclax and HMAs has demonstrated its potential for elderly patients who are not suitable for intensive chemotherapy, showcasing enhanced efficiency and safety. The combination of Venetoclax and HMAs may synergistically exert anti-tumor effects by upregulating the expression of pro-apoptotic proteins NOXA and PUMA, while inhibiting the activity of pro-survival proteins MCL1 and BCL-XL, thereby facilitating apoptosis in AML tumors (12). The combination therapy of azacitidine and venetoclax demonstrated a remarkable complete remission rate of 71% in patients who were ineligible for intensive chemotherapy, as evidenced by a published report (13). Unfortunately, the patient in this study did not achieve remission from either intensive chemotherapy or the combination of venetoclax and HMAs. Meanwhile, due to a progressively deteriorating physical condition, the patient was unable to tolerate subsequent intensive chemotherapy.

The field of CAR-T therapy has made significant advancements in the treatment of hematological malignancies (14–16). Recent research findings have demonstrated that CAR-T therapy exhibits comparable efficacy in both elderly and younger patients, without any observed increase in adverse effects, suggesting that CAR-T therapy should not be excluded as a treatment option for elderly patients (17, 18). Considering the absence of any uncontrollable toxicities in our previous clinical trial on CLL1 CAR-T therapy, we decided to prioritize the employment of CLL1 CAR-T therapy for the treatment of this elderly patient, accompanied by comprehensive supportive care. Fortunately, the CLL1 CAR-T cells effectively eradicated the majority of tumor cells, as flow cytometry analysis revealed only 2.58% of karyocytes were identified as abnormal myeloid progenitor cells. However, the CAR-T cells also demonstrated enhanced recognition and elimination of neutrophils due to their heightened expression of CLL1 on the cell surface, resulting in irreversible granulocytopenia. Therefore, allo-HSCT was employed to simultaneously enhance efficacy and promote hematopoiesis recovery. Furthermore, our previous reports and the research conducted by Miao et al. have consistently demonstrated the efficacy and safety of allo-HSCT as a consolidation and bridging therapy regimen subsequent to CLL1 CAR-T therapy (8, 19). The pre-conditioning regimen, particularly the administration of antihuman thymocyte globulin, effectively eradicated CAR-T cells prior to stem cell infusion. Consequently, we observed no evidence of recognition and elimination of donor-targeted CAR-T cells against neutrophils. Furthermore, the engraftment time for granulocytes in this patient was comparable to that of patients who did not undergo CLL1 CAR-T cell therapy, with no significant differences noted. Despite the patient having experienced a series of complications associated with HSCT, such as infection and GVHD, we successfully resolved these issues and significantly improved the patient’s quality of life.

In summary, despite the successful improvement in prognosis for elderly patients achieved through low-intensity chemotherapy comprising venetoclax and HMAs, there remains a pressing need to explore novel treatment options for patients who exhibit resistance to current therapies. In this paper, we propose an innovative treatment strategy that combines CLL1 CAR-T therapy with allo-HSCT, which has demonstrated remarkable tolerability and promising benefits even in elderly patients as reported herein.

## Data Availability

The raw data supporting the conclusions of this article will be made available by the authors, without undue reservation.
